# DESI-MSI-Based Multi-Organ Distribution Mapping of Psilocin in Zebrafish

**DOI:** 10.3390/molecules31122143

**Published:** 2026-06-18

**Authors:** Mengxuan Dong, Yi Zhang, Manzhu Cao, Tong Shi, Liqin Li, Xingxing Zong, Chen Wang

**Affiliations:** State Key Laboratory of Chemistry for NBC Hazards Protection, Beijing 102205, China; 15154127769@163.com (M.D.); zhangyi2017yaoxue@163.com (Y.Z.); cao09221@163.com (M.C.); tong198282@126.com (T.S.); llq969696@126.com (L.L.)

**Keywords:** psilocybin, psilocin, DESI-MSI, zebrafish, neurotoxicity, spatial distribution

## Abstract

Psilocybin, a psychedelic drug with reported anxiolytic and antidepressant potential, is rapidly metabolized to its active metabolite psilocin. However, a lack of adequate toxicity studies and tissue distribution studies currently restricts its development and application. This study combined behavioral assays in zebrafish with desorption electrospray ionization mass spectrometry imaging (DESI-MSI) to systematically evaluate the acute neurotoxicity of psilocybin and characterize the in vivo spatial distribution of its active metabolite, psilocin. The novel tank test was used to evaluate zebrafish following a 4 h exposure to psilocybin at three different doses (20, 40, and 80 μM; *n* = 6 per group). Statistical analysis of the data was performed using ANOVA. Behavioral analyses revealed that exposure to psilocybin induced pronounced neurobehavioral alterations, including hyperactivity and disrupted swimming patterns, as evidenced by significant increases in the number of zone transitions and shuttle frequency. We established a DESI-MSI-based method for quantitative mapping and visualization of psilocin in zebrafish tissues. Methodological validation indicated that a linear relationship between ion intensity, spotted amount (R^2^ = 0.9947), and reproducibility (RSD < 15%) is suitable for quantitative analysis of psilocin in zebrafish tissues. Spatial distribution maps showed that following continuous exposure for 4 h, psilocin was widely distributed across multiple tissues, such as the eye, brain, heart, liver, and kidney, with marked accumulation in the brain and the periportal regions of the liver. Relative psilocin signal intensity revealed a dose-dependent increase in tissue drug levels. The dose-dependent increase in both behavioral hyperactivity and brain psilocin levels points to a consistent relationship, in line with a central site of action. Collectively, these findings demonstrate that DESI-MSI provides a visual and efficient strategy for studying drug distribution in biological tissues from exposed animals. The neurobehavioral toxicity phenotypes and distinct tissue distribution patterns of psilocin uncovered in this study offer critical insights into the biological effects and potential risks of this psychoactive substance.

## 1. Introduction

Psilocybin and its active metabolite psilocin are undergoing a paradigm shift—from classification as illicit drugs to recognition as revolutionary therapeutic agents [1,2,3,4]. Psilocybin is a phosphorylated tryptamine that is rapidly dephosphorylated to psilocin upon ingestion. Psilocin crosses the blood–brain barrier and acts as a potent partial agonist at the 5-HT2A receptor, eliciting profound perceptual alterations [5,6,7,8]. In terms of clinical potential, multiple recent randomized controlled trials and systematic reviews have demonstrated that psilocybin combined with psychotherapy produces significant and sustained efficacy in treatment-resistant depression, end-of-life anxiety, substance use disorders and post-traumatic stress disorder [9,10,11,12]. For instance, a single high dose of psilocybin in cancer patients significantly reduced depression and anxiety levels for up to six months [13]. However, the hallucinogenic toxicity of psilocybin cannot be overlooked: high doses may precipitate acute psychotic symptoms, panic attacks, perceptual abnormalities, and, in rare cases, the risk of hallucinogen-persisting perception disorder (HPPD) [14,15]. This “double-edged sword” profile underscores the urgent need for systematic investigations into the pharmacological properties, in vivo behavior, and safety profiles of both psilocybin and psilocin [16].

Among various animal models, the zebrafish (*Danio rerio*) has emerged as a key platform for neurotoxicology and neuropsychopharmacology, owing to its highly conserved genome, optically transparent embryos, short reproductive cycle, and high-throughput screening capability [17,18,19]. In recent years, zebrafish have been increasingly used in hallucinogen research, particularly for evaluating the behavioral effects of serotonergic drugs. Studies have shown that psilocybin significantly alters the locomotor patterns of zebrafish larvae, including increased rapid swimming and reduced erratic swimming following stress—behavioral changes that closely mirror the anxiolytic and stimulating effects observed in humans [17]. An existing high-throughput behavioral analysis based on computer vision and motion tracking enables accurate capture of subtle locomotor changes in zebrafish within their microenvironment [17,19]. This method offers high sensitivity, reproducibility, and broad applicability, making it suitable not only for acute toxicity screening but also for mechanistic studies and dose–response analyses, thereby providing a powerful tool for behavioral assessment of psilocybin and psilocin.

In the field of analytical chemistry, methods for detecting psilocybin and psilocin have advanced considerably. Liquid chromatography–tandem mass spectrometry (LC-MS/MS) is now the gold standard for quantifying psilocybin and psilocin in plant materials (e.g., mushrooms, mycelium), offering exceptional sensitivity and specificity [20,21,22]. Regarding in vivo pharmacokinetics, existing studies have characterized the pharmacokinetic profile of psilocybin in humans, including its rapid conversion to psilocin, dynamic changes in plasma concentration, and correlation with clinical effects [18,23,24]. Madsen and colleagues used Positron Emission Computed Tomography (PET) imaging to demonstrate that plasma psilocin concentration is positively correlated with 5-HT2A receptor occupancy and the intensity of subjective hallucinogenic experiences [25]. However, current in vivo studies have largely focused on biofluids such as plasma and urine. A systematic, in-depth investigation into the tissue distribution of psilocybin and psilocin across organs—including brain, liver, kidney, and heart—and their inter-tissue transport mechanisms remains lacking. This absence of tissue distribution data limits our understanding of their targets of action, toxicological mechanisms, and inter-individual variability.

Desorption electrospray ionization mass spectrometry imaging (DESI MSI) is an ambient ionization mass spectrometry imaging technique that combines electrospray ionization (ESI) with desorption ionization [26]. As a label-free imaging modality, DESI MSI enables the generation of chemical distribution maps directly from the surface of biological samples without the need for complex sample preparation [27]. Since its introduction as an imaging technique in 2005, DESI MSI has been widely developed and applied for the differentiation and diagnostic characterization of various pathological tissue types [28,29]. The technical principle of DESI-MSI is that a charged solvent spray generated by the DESI probe is directed at supersonic speed onto the surface of a sample mounted on a movable stage. Upon impact, the charged solvent microdroplets desorb analyte molecules from the surface and extract them into secondary microdroplets. These secondary microdroplets then undergo gas-phase ion formation under ambient atmospheric pressure, and the resulting ions are transmitted into a mass spectrometer for detection. Simultaneously, two-dimensional spatial scanning is achieved by controlling the movement of the sample stage, thereby generating a chemical distribution map of molecules across the sample surface [26,28,30]. This technique offers high spatial resolution (typically 50–100 μm), multiplexed detection, and three-dimensional reconstruction capabilities. It has been successfully applied to visualize drug distribution in animal tissues, including liver, brain, and skin [31,32]. In pharmacokinetic studies, DESI-MS can directly reveal the spatial heterogeneity of drug and metabolite distribution within the tissue microenvironment, uncovering information lost in conventional homogenization-based approaches [19,33,34]. Applying DESI-MS to map the in vivo distribution of psilocin is not only feasible but also highly innovative, and is expected to generate the first whole-organ to subcellular distribution maps of these two compounds in zebrafish.

This article aims to establish a testing method for the hallucinogenic activity of psilocybin based on a zebrafish model coupled with high-throughput behavioral assessment, and to integrate DESI-MS imaging for the first systematic investigation of the tissue distribution of psilocin in zebrafish. This study will elucidate the spatiotemporal distribution characteristics of psilocin in key organs, including the brain, liver, and kidney, and define its differential distribution across these tissues, thereby providing critical data for mechanistic insights into psilocybin and for advancing neuropharmacology. Furthermore, it will drive the innovative application of in situ mass spectrometry imaging in the development of psychotropic drugs.

## 2. Results and Discussion

### 2.1. Behavioral Assays

**Figure 1:**
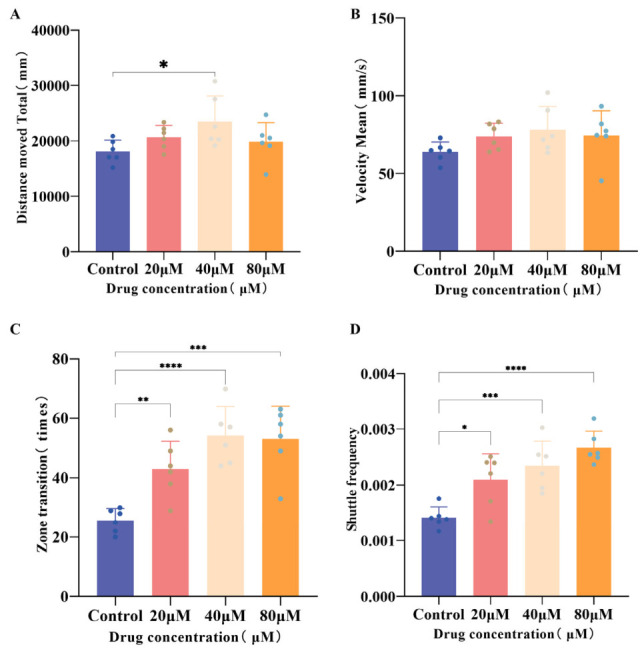
Neurotoxic effects of psilocybin on adult zebrafish. (**A**) Distance moved Center-point Total; (**B**) Velocity Center-point Mean; (**C**) Zone transitions; (**D**) Shuttle frequency; *n* = 6; * *p* < 0.05, ** *p* < 0.01, *** *p* < 0.001, **** *p* < 0.0001.

**Figure 2:**
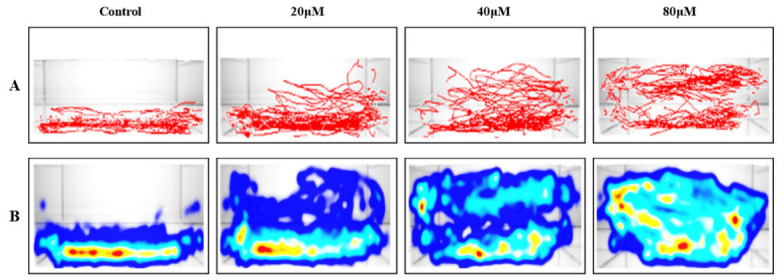
Zebrafish neurotoxicity experiment-trajectory plot and heatmap of the novel tank test. (**A**) Trajectory Plot; (**B**) Heatmap.

The novel tank test revealed that psilocybin exposure induced neurobehavioral alterations in zebrafish, characterized by hyperactivity and erratic swimming. Compared with the control group, the total distance moved (Figure 1A) and velocity mean (mm/s) (Figure 1B) in the treatment groups initially increased and then decreased. However, one-way ANOVA revealed no statistically significant differences (*p* = 0.0658 for total distance moved; *p* = 0.2418 for velocity mean). In contrast, the number of zone transitions (Figure 1C) and shuttle frequency (Figure 1D) showed a concentration-dependent increase. Specifically, zone transitions increased by approximately 167.53%, 211.04% and 206.49% across the treatment groups, while horizontal shuttling frequency increased by approximately 147.51%, 165.17% and 187.48% respectively. One-way ANOVA followed by Tukey’s post hoc test revealed an effect of psilocybin concentration on zone transitions (*p* < 0.0001) and on shuttling frequency (*p* < 0.0001). The trajectory plots (Figure 2A) and heatmaps (Figure 2B) visually corroborated these behavioral findings, demonstrating that increasing psilocybin concentrations led to progressively more active and disorganized swimming patterns. The aberrant behaviors induced by psilocybin in the novel tank test—namely hyperactivity and erratic swimming—are consistent with findings from a high-resolution behavioral tracking study, which reported that acute psilocybin treatment increases rapid swimming and induces irregular locomotor patterns [17]. However, unlike the pronounced behavioral shifts observed in that study, our data showed that the total distance moved and average swimming speed exhibited a biphasic trend without reaching statistical significance [35]. This discrepancy may reflect concentration- and time-dependent differences in psilocybin’s effects. Notably, concentration-dependent increases in zone transitions and horizontal shuttling frequency align with earlier behavioral phenotyping studies, which indicated that activation of the 5-HT1A receptor may promote sociability and exploratory behavior [25]. Human clinical studies have employed functional brain imaging techniques (Magnetoencephalography and Electroencephalography, MRI/EEG) to monitor changes in brain network connectivity and have assessed alterations in mental state, as well as improvements in depressive and anxiety symptoms, using subjective experience reports and clinical scales [36]. Human studies have also demonstrated time-dependent characteristics, with scans at 20, 40, and 70 min respectively indicating that changes in brain connectivity are time dependent. Notably, a single high dose of 25 mg in human clinical trials produced rapid and sustained therapeutic effects lasting up to 12 months [37,38].

### 2.2. Establishment and Optimization of a Detection Method for Psilocin Using DESI-MSI

#### 2.2.1. Optimization of DESI-MSI Parameters

**Figure 3:**
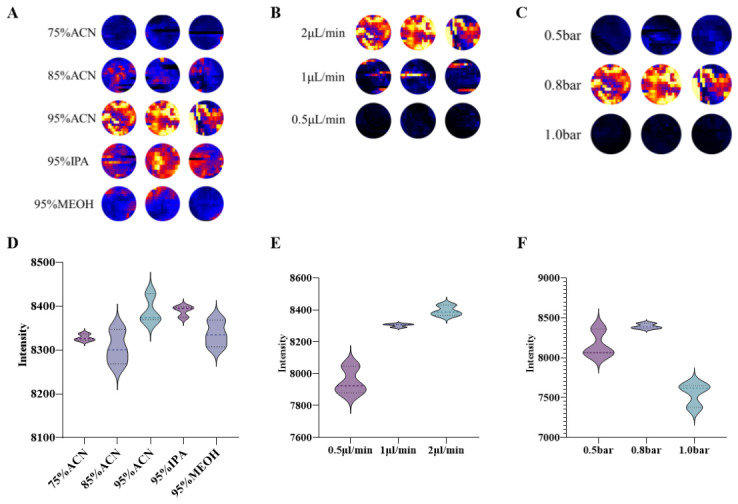
Optimization of DESI-MSI detection conditions for psilocin. (**A**) Representative DESI-MS imaging of psilocin during mobile phase optimization; (**B**) Representative DESI-MS imaging of psilocin during flow rate optimization; (**C**) Representative DESI-MS imaging of psilocin during nitrogen pressure optimization; (**D**) Quantitative results of the psilocin standard during mobile phase optimization; (**E**) Quantitative results of the psilocin standard during flow rate optimization; (**F**) Quantitative results of the psilocin standard during nitrogen pressure optimization.

Based on a combination of literature review and experimental data, we further analyzed the influence of key parameters on signal quality in DESI MSI. The choice of spray solvent is a critical determinant of ionization efficiency. As highlighted in a comprehensive review, solvent polarity and volatility directly affect surface wettability and analyte solubility, thereby modulating ion transfer efficiency [27,39]. Our experimental results show that a 95% acetonitrile (ACN) system with low water content (Figure 3A,D) yields higher ion intensity and sharper imaging boundaries compared to methanol (MeOH) or isopropanol (IPA) systems. This is consistent with its lower surface tension and higher volatility, which facilitate more effective analyte extraction from the tissue surface, rapid dissolution, and efficient ejection into the vacuum [39]. Regarding the effect of spray flow rate, previous work has established an inverse relationship between flow rate and droplet size [19]. Experimental results (Figure 3B,E) showed that signal intensity increased when the flow rate was raised from 1.0 μL/min to 2.0 μL/min. This is likely due to unstable droplet formation or deformation of the electrospray cone at lower flow rates, which compromises the charging efficiency of analytes [30]. Although lower flow rates can reduce solvent consumption and improve spatial resolution, we determined that a flow rate of 2.0 μL/min represents the optimal balance to achieve high detection sensitivity in this study. Nitrogen pressure is another key factor governing spray dynamics. Literature indicates that excessive gas pressure leads to over-nebulization and splashing, ultimately reducing signal stability [40]. Our experimental data (Figure 3C,F) confirm that a nitrogen pressure of 0.8 bar provides sufficient spray momentum to maintain a stable electrospray cone without blowing the sample solvent off the tissue surface, thereby ensuring optimal detection sensitivity.

#### 2.2.2. Methodological Validation for Quantitative DESI-MSI Analysis

- Specificity

**Figure 4:**
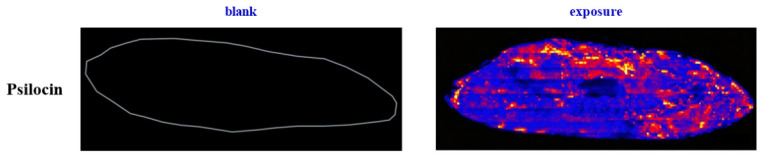
Imaging of psilocin in zebrafish sections.

The specificity of Psilocin ([M + H]^+^ 205.1341) in DESI-MSI was assessed by comparing images obtained from blank zebrafish sections and actual zebrafish samples. No significant endogenous substances or impurities interfered with the detection of analytes in the positive ion mode (Figure 4).

- Calibration curve and performance

**Figure 5:**
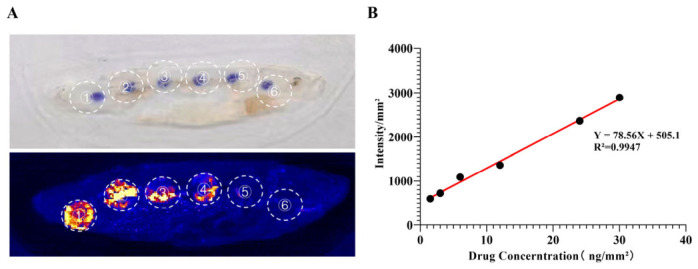
Methodological evaluation of DESI-MS imaging: calibration curve. (**A**) Images of psilocin spots (six parallel points) on a glass slide, along with psilocin detected in the zebrafish spine and the ion at *m*/*z* 205.1341 as measured by DESI-MS imaging. (**B**) Calibration curve generated by micros potting psilocin standards at concentrations of ① 30.0 ng/mm^2^, ② 24.0 ng/mm^2^, ③ 12.0 ng/mm^2^, ④ 6.0 ng/mm^2^, ⑤ 3.0 ng/mm^2^ and ⑥ 1.5 ng/mm^2^ onto the spinal region of zebrafish tissue sections. The correlation coefficient was 0.9947.

In this study, we successfully established a semi-quantitative method for detecting psilocin in zebrafish tissue sections. The method exhibited excellent linearity over a concentration range of 1.5–30.0 ng/mm^2^ (R^2^ > 0.99, Figure 5), meeting the stringent requirements for bioanalytical sample analysis.

- Matrix effects, Accuracy, and Precision

Notably, the matrix effects for psilocin ranged from 90.50% to 113.35%, with relative standard deviations (RSDs) below 10.28% (Table 1), indicating acceptable matrix effects and method reliability. The RSDs of the accuracy and precision tests were 1.43–17.87% and 4.81–14.97% respectively (Table 1), indicating good precision and accuracy of the instrument. Collectively, this DESI-MS analytical method proves to be sensitive, accurate, and reliable.

### 2.3. Visualization and Quantitative Analysis of Psilocin Distribution in Zebrafish

**Figure 6:**
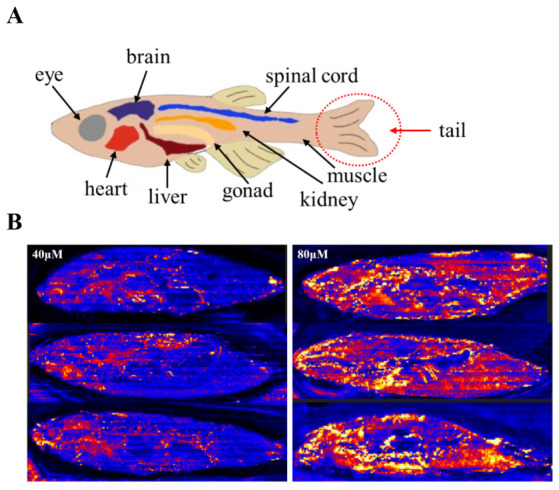
DESI-MSI analysis of zebrafish sections. (**A**) Distribution in different organs of zebrafish and mass spectrometry scanning images. (**B**) Representative DESI-MSI images of zebrafish tissue sections after exposure to different psilocybin concentrations.

**Figure 7:**
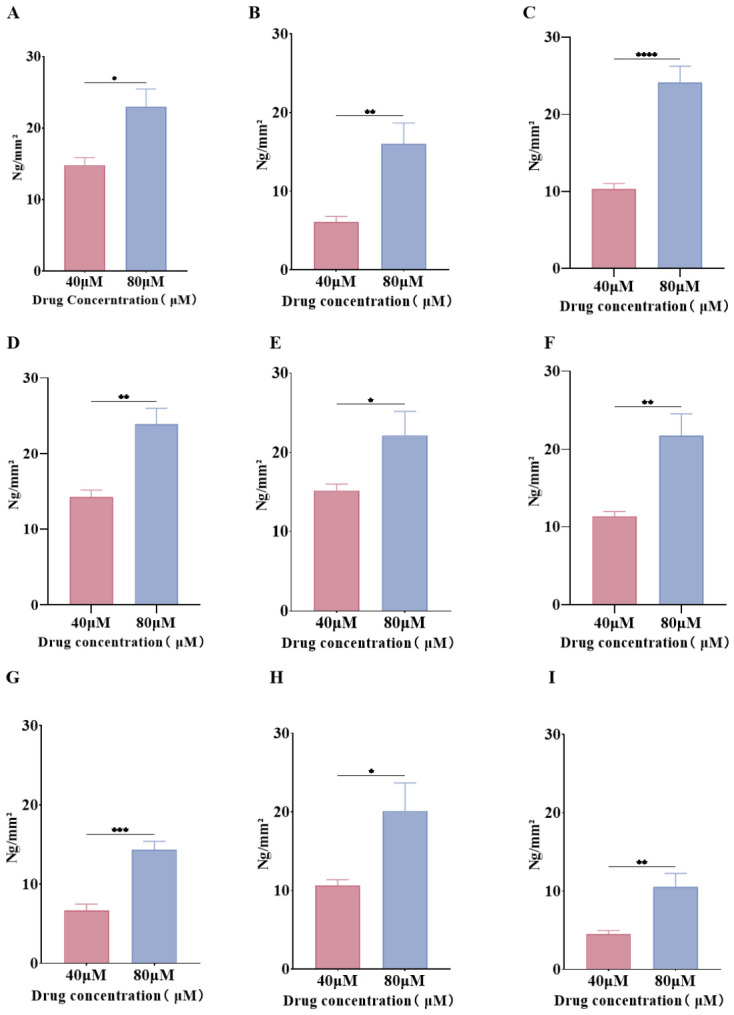
Concentration distribution in various tissues of zebrafish. (**A**) Eye; (**B**) Brain; (**C**) Heart; (**D**) Liver; (**E**) Kidney; (**F**) Gonad; (**G**) Spinal cord; (**H**) Muscle; (**I**) Tail. For panels with *n* = 6, * *p* < 0.05, ** *p* < 0.01, *** *p* < 0.001, **** *p* < 0.0001.

DESI-MSI was used to characterize the spatial distribution of psilocin in zebrafish. Nine tissues—eye, brain, heart, liver, kidney, gonad, spinal cord, muscle, and tail—were selected as regions of interest (ROIs) for quantitative analysis of relative signal intensities. As shown in Figure 6, after 4 h of continuous exposure, psilocin accumulated in all the aforementioned tissues, with predominant enrichment in the brain and periportal regions of the liver. Raw signal intensities were normalized to an internal standard (Dimethyltryptamine, DMT, 500 ng/mL) added to the spray solvent. In the 80 μM treatment group, the normalized signals were 232.91%, 189.31%, 214.03%, 145.50%, 192.94%, 167.30%, 233.83%, 154.98% and 262.1% of those in the 40 μM group for the eye, brain, heart, liver, kidney, gonad, spinal cord, muscle, and tail, respectively (Figure 7). Across the nine tissues examined, psilocybin signal intensity in the 80 μM group was significantly higher than that in the 40 μM group, with statistically significant differences observed between groups. The most pronounced differences were found in heart and spinal cord tissues (*p* < 0.001), followed by brain, liver, gonad, and tail tissues (*p* < 0.01) and eye, kidney, and muscle tissues (*p* < 0.05). These results mirror the concentration-dependent trend observed in the behavioral assays.

Based on the results of this study, psilocin exhibits a distinct tissue enrichment profile in zebrafish. Following four hours of continuous exposure, the drug molecules predominantly accumulate in the brain and the perihepatic regions. This distribution pattern aligns with the known blood–brain barrier permeability and metabolic characteristics of psilocin. Moreover, the accumulation of the drug in tissues such as the eyes, heart, and kidneys increases in a dose-dependent manner. The use of desorption electrospray ionization mass spectrometry imaging (DESI-MSI) to map drug distribution in zebrafish has been widely validated as an efficient approach [19,34]. In this study, the observed cerebral enrichment of psilocin is highly consistent with the assessment of its blood–brain barrier permeability in the zebrafish model, further confirming the utility of zebrafish as a predictive model for the distribution of central nervous system drugs. Furthermore, the significant, dose-dependent increase in psilocin levels in the eyes, heart, and kidneys indicates a dose-responsive absorption and distribution profile, consistent with its pharmacokinetic behavior in preclinical studies [23]. Collectively, these results demonstrate that DESI-MSI can successfully delineate the spatial distribution of psilocin in zebrafish, highlighting the reliability of this technique for in vivo drug distribution studies.

## 3. Materials and Methods

### 3.1. Materials and Reagents

LC-MS grade methanol (Cat# A456-4), LC-MS grade acetonitrile (Cat# A451-4), and formic acid (Cat# A117-50) were purchased from Thermo Fisher Scientific (Shanghai, China). Psilocybin and psilocin reference standards (purity: reference standard grade) were purchased from Shanghai Yuansi Bio-technology Co., Ltd. (Shanghai, China). Positive ion anti-detachment glass slides (Cat# 4951PLUS-001E) were purchased from Epredia (Beijing, China).

### 3.2. Experimental Animals

Adult zebrafish (total length: 3–4 cm) were fed commercial brine shrimp twice daily. Prior to experiments, fish were acclimated for at least two weeks in aerated culture water maintained at 28 ± 1 °C, with a pH of 7.0–8.5, dissolved oxygen > 80% air saturation, and a hardness of 200 ± 25 mg/L (as CaCO_3_). The reconstituted water contained 2 mmol/L Ca^2+^, 0.5 mmol/L Mg^2+^, 0.75 mmol/L Na^+^, and 0.074 mmol/L K^+^. A 14 h light:10 h dark photoperiod was maintained throughout. Water was continuously aerated and dechlorinated. All animal procedures with animals performed in this study were approved by State Key Laboratory of Chemistry for NBC Hazards Protection under permission NO. LAE-2025-005-003 (Beijing, China) [19,41,42].

A stock solution of psilocybin (10 mM/L in DMSO) was quantitatively diluted in fish culture water to achieve the target test concentrations. Adult zebrafish were randomly assigned to four experimental groups: a control group (Con) and three psilocybin exposed groups—low-dose (L, 20 μM/L), medium-dose (M, 40 μM/L), and high-dose (H, 80 μM/L) with six fish per group (Twenty-four zebrafish were numbered 1 to 24 and then randomly divided into four groups of six using Excel. A random number was generated for each fish, and the sorted list was sequentially allocated to the four groups). Following a two-week acclimation period, zebrafish were continuously exposed to psilocybin for 4 h. After psilocybin treatment, zebrafish were subjected to behavioral tests and DESI MSI experiments. Euthanasia was performed by hypothermic shock at the conclusion of the behavioral tests and during the preparation of frozen sections. The tasks of drug administration and daily animal care, outcome assessment, and statistical analysis of data were performed by different experimenters, respectively, ensuring no mutual interference.

### 3.3. Neurotoxic Effects of Psilocybin on Adult Zebrafish

#### Behavioral Assays

Based on our preliminary findings in zebrafish, we selected exposure concentrations of 20, 40, and 80 μM (μmol/L) of psilocybin. Prior to behavioral testing, groups of six zebrafish were immersed in 1 L glass beakers containing the respective psilocybin solutions for 4 h. Control fish were exposed to drug-free water under otherwise identical conditions. Behavioral assays were conducted between 11:00 a.m. and 3:00 p.m., with the water temperature in the test tank adjusted to match that of the housing system. All behavioral recordings commenced after a 10 min acclimation period.

The novel tank test was used to assess zebrafish behavior 4 h after treatment with different doses of psilocybin. The novel tank (3.75 L; 25 cm (L) × 10 cm (W) × 15 cm (H)) was filled with water and externally divided into two equal horizontal zones (upper and lower) using markings on its outer wall. As previously described, a side-mounted camera recorded zebrafish behavior to quantify total distance moved (mm), mean swimming speed (mm/s), number of zone transitions, and shuttling rate.

Trajectories were analyzed using EthoVision XT 7 software. Video was recorded in MPEG1 (Moving Pictures Experts Group1) format at a maximum sampling rate of 30 fps using an auto-focus 2.0 MP USB webcam placed 20 cm from the tank front and connected to a laptop. Recorded videos were analyzed with EthoVision XT 7 as previously described. The arena was calibrated for each fish, and the calibration axis was placed at the tank center to designate the origin (0.0). Tracking data were exported as raw data to spreadsheets. Exported data were evaluated for consistency by trained observers blinded to treatment groups.

### 3.4. Establishment and Optimization of a Detection Method for Psilocin Using DESI-MSI

#### 3.4.1. Establishment of the DESI-MSI In Situ Detection Method

- Sample preparation

To prepare the matrix-free sample, 1 μL of a 100 μg/mL psilocin solution (dissolved in methanol: water, 1:1, *v*/*v*) was spotted onto the designated spot of a sample plate and left to dry at room temperature for 3–5 min until the solvent had completely evaporated. For the matrix-coated sample, blank zebrafish sections (from untreated animals) were removed from the freezer and allowed to equilibrate at room temperature for 5–10 min to evaporate surface moisture. Subsequently, 1 μL of the same psilocin solution (100 μg/mL in methanol: water, 1:1) was spotted onto the same matrix-coated area of the blank section and air-dried for 3–5 min.

- Spray solution preparation

A 20 mL solvent mixture of methanol and water (95:5, *v*/*v*) was prepared, sonicated, and filtered. Leucine enkephalin was added to the solvent at a concentration of 50 pg/mL for mass calibration (ESI^+^: 556.2771; ESI^−^: 554.2615). DMT was included as an internal standard at a concentration of 500 ng/mL.

- Parameter settings

The solvent spray parameters were set as follows: flow rate of 2.0 μL/min, DESI gas pressure of 0.80 MPa. The solvent was introduced into the injector and allowed to equilibrate until a stable spray was achieved. The DESI moving stage was configured with the following parameters: sprayer-to-slide distance of 3 mm, sprayer-to-transfer tube distance of 4 mm, transfer tube-to-slide distance of 1 mm, sprayer angle of 65°, sampling resolution of 100 μm × 100 μm, and sampling speed of 500 μm/s. The capillary voltage was set to 0.7 kV, sampling cone voltage to 40 V, source temperature to 150 °C, and transfer tube temperature to 250 °C. After stabilization of the solvent spray, signal response intensities were confirmed in positive-ion and negative-ion modes using Waters (Milford, MA, USA) designated red signal pen (ESI^+^: 443.2335) and black signal pen (ESI^−^: 666.0600), respectively.

- Data acquisition and processing

Samples were placed on the DESI MSI moving stage. The region of interest for DESI MSI data acquisition was delineated according to the sample position, and data were acquired in positive ion mode to obtain raw files (.raw format). Using the “Process” module of HDI software (version 1.7, Waters, Milford, MA, USA), the .raw files acquired by the QTof mass spectrometer were converted into a data matrix (.txt format). Mass calibration was performed at intervals of 5 min using leucine enkephalin as the lock mass compound.

Calibrated .txt files were opened in the “Analysis” module of HDI software. Ion images for psilocin ([M + H]^+^, *m*/*z* 205.1341) and DMT ([M + H]^+^, *m*/*z* 189.1392) were extracted. Regions of interest (ROIs) were defined by co registration with the optical image of the brain section. Within each ROI, the response intensities of psilocin and DMT were obtained, and the relative response was calculated as the psilocin-to-DMT intensity ratio.

#### 3.4.2. Optimization of the DESI MSI Detection Method

The composition of the mobile phase is critical for achieving rapid spatially resolved targeted detection. Therefore, different mobile phase compositions were tested, including 75% acetonitrile (ACN), 85% ACN, 95% ACN, 95% isopropanol (IPA), and 95% methanol (MeOH), and the signal responses were evaluated to optimize the mobile phase system. In addition, imaging performance was compared at flow rates of 0.5 μL/min, 1.0 μL/min, and 2.0 μL/min. Nitrogen pressure was also optimized at 0.5 bar, 0.8 bar, and 1.0 bar to obtain the best spray conditions.

#### 3.4.3. Methodological Validation

- Specificity

The method specificity was validated by assessing the interference of endogenous substances with the detection of the target compound. To this end, zebrafish tissue sections from a blank control group and a treatment group (administered with 40 μM of the psilocybin) were examined. All samples were subjected to identical pretreatment procedures prior to analysis. By comparing the mass spectrometry imaging (MSI) maps obtained from the two groups, potential interference from endogenous substances was evaluated, thereby confirming the method’s specificity.

- Calibration curve and performance

Preparation of spotted samples at different concentrations on blank brain sections: Blank sections (from zebrafish not subjected to drug treatment) were removed from the freezer and allowed to dry at room temperature for 5–10 min to evaporate surface moisture. Then, 0.3 μL psilocin solutions prepared in MeOH:water (1:1, *v*/*v*) at concentrations of 5 μg/mL (1.5 ng/mm^2^), 10 μg/mL (3.0 ng/mm^2^), 20 μg/mL (6.0 ng/mm^2^), 40 μg/mL (12.0 ng/mm^2^), 80 μg/mL (24.0 ng/mm^2^), and 100 μg/mL (30.0 ng/mm^2^) were spotted onto the same matrix region of consecutive blank sections. The spots were left to stand for 3–5 min until the solvent had evaporated. The samples were subsequently analyzed using the DESI MSI method for psilocin detection.

- Matrix effects, Accuracy, and Precision

Matrix effects, precision and accuracy were evaluated at three levels (1.5–30.0 ng/mm^2^, *n* = 3). For matrix effects, Matrix-free samples were prepared by directly spotting 0.3 μL of standard solution onto the sample plate, followed by analysis using desorption electrospray ionization mass spectrometry (DESI-MS). Matrix-containing samples were prepared by spotting 0.3 μL of standard solution onto blank zebrafish tissue sections, which were then analyzed by DESI-MS. The matrix effect was assessed by calculating the ratio A/B, where A represents the peak intensity ratio of the analyte to its corresponding internal standard in the matrix-containing samples, and B represents the same ratio in the matrix-free samples. Accuracy and precision were determined by comparing the measured values with the nominal concentrations at each concentration level.

### 3.5. Visualization of the Spatial Distribution of Psilocin in Zebrafish

Cryosections of zebrafish were prepared for this study. Two exposure concentration groups were set (40 μM and 80 μM, *n* = 6) The zebrafish were anesthetized in ice-cold water for 1 min, rinsed three times with phosphate-buffered saline (PBS), and the caudal fins were removed. The samples were then embedded in optimal cutting temperature (OCT) compound and stored at −80 °C. Thirty minutes before sectioning, the samples were transferred to a cryostat to equilibrate to the chamber temperature. Under −22 °C conditions, the whole zebrafish were sectioned into 40 μm thick cryosections. Following sectioning, targeted scanning analysis was performed on the entire fish.

A Synapt XS high-resolution mass spectrometer (Waters, Milford, MA, USA) equipped with a DESI ion source was used for in situ analysis of psilocin spatial distribution in zebrafish in positive ion mode. Target ions included psilocin (*m*/*z*: 205.1341) and the internal standard DMT (*m*/*z*: 189.1392). The DESI-MSI solvent system was acetonitrile–water (95:5, *v*/*v*) at a flow rate of 2.0 μL/min; nitrogen pressure was 0.80 bar, and spray voltage was 0.70 kV. The spatial scanning resolution for zebrafish tissue sections was set to 100 × 100 μm.

Using HDI V1.7 software, nine tissues (eye [E], brain [B], heart [H], liver [L], kidney [K], gonad [Go], spinal cord [S], muscle [M], and tail [T]) were selected as regions of interest (ROIs). Relative distribution levels of psilocin were assessed by calculating its relative intensity (ratio to DMT ion intensity) in each ROI.

### 3.6. Data Analysis

Data were statistically analyzed and illustrated by GraphPad Prism 10.1.2. Pairwise comparisons were analyzed with Student’s t tests, while multi group datasets underwent one way analysis of variance (ANOVA), followed by Dunnett’s post hoc test for multiple comparisons. Statistical significance was defined as *p* < 0.05, with all values reported as mean ± standard deviation (SD).

## 4. Conclusions

This study investigated the acute neurotoxic effects of psilocybin in zebrafish and presents a novel, efficient, and systematic approach for mapping the in vivo spatial distribution of its metabolite, psilocin. Our behavioral assays revealed that psilocybin induces hyperactivity and concentration-dependent increases in zone transitions, while DESI-MSI showed predominant psilocin accumulation in the brain and periportal liver regions. The method also enabled spatial localization of compounds within entire zebrafish sections. Furthermore, DESI-MSI successfully facilitated the quantitative analysis and spatial mapping of psilocin in zebrafish. These findings highlight the potential risks associated with psilocybin exposure and support the need for further toxicological evaluation. This analytical strategy may serve as a reference for assessing the toxicity of other tryptamine-based compounds.

## 5. Patents

The research results reported in this paper have led to one patent “A method for in vivo visual detection of psilocybin metabolites.”

## Figures and Tables

**Table 1:** Performance of psilocin in zebrafish (*n* = 3).

Compound	Concentration (ng/mm^2^)	Accuracy (100%)	Precision (100%)	Matrix Effects	
				Mean ± SD (%)	RSD (%)
Psilocin	1.5	17.87	13.64	113.35 ± 11.10	9.79
	12	9.24	14.97	90.50 ± 8.79	9.71
	30	1.43	4.81	105.81 ± 10.87	10.28

## Data Availability

The original contributions presented in this study are included in the article. Further inquiries can be directed to the corresponding authors.
